# Measuring the Rider–Horse Dyad Across Olympic Equestrian Disciplines: A Scoping Review of Methodological Gaps and Priorities for Show Jumping

**DOI:** 10.3390/ani16193076

**Published:** 2026-09-30

**Authors:** Hui Huang, Zhongjie Yan, Xinrong Chen

**Affiliations:** 1 School of Physical Education, Shanghai University, 99 Shangda Road, Baoshan District, Shanghai 200444, China; hh201688202408@163.com (H.H.); yanzhongjie007@aliyun.com (Z.Y.); 2 College of Biomedical Engineering, Fudan University, 130 Dongan Road, Xuhui District, Shanghai 200032, China

**Keywords:** rider–horse coordination, biomechanics, equine welfare, show jumping, inertial measurement unit (IMU), surface electromyography (sEMG)

## Abstract

(1) Background: Rider–horse coordination underpins equestrian performance; yet, its definition, quantitative indicators, and measurement methods remain inconsistent across Olympic disciplines. This scoping review mapped the constructs, metrics, and measurement approaches in dressage, show jumping, eventing, and transferable equestrian contexts, with show jumping as the principal application context. (2) Methods: Following PRISMA-ScR guidance and a Population–Concept–Context framework, Web of Science Core Collection, Scopus, and PubMed were searched from database inception to 10 September 2026. Evidence was charted by discipline, measurement domain, measurement configuration, and synchronisation status. (3) Results: A total of 121 evidence sources were included. Among 108 empirical or methodological studies, 47 were kinematic/biomechanical, 23 performance/statistical, 15 behavioural/welfare-related, 13 kinetic/pressure/interface, and 10 neuromuscular/physiological. Only 12 used synchronised rider–horse time-series or event-based measurements, and none of the show-jumping studies met the strict synchronisation criterion. Coordination was commonly inferred from single-partner signals, interface measures, or aggregate performance outcomes. (4) Conclusions: The key limitation is not sensor availability, but the lack of validated dyadic methods. Future show-jumping studies should use common time bases, predefined biomechanical events, paired variables, and prospective validation, while integrating behavioural and clinical evidence when interpreting welfare-related outcomes.

## Simple Summary

Rider–horse coordination is fundamental to equestrian performance and is particularly important in show jumping, where take-off, flight, and landing occur within very short time periods. However, there is currently no consensus on the definition, quantitative indicators, or measurement methods for rider–horse coordination. This scoping review systematically examined equestrian studies in dressage, show jumping, eventing, and other disciplines with methodological transferability, aiming to identify how rider–horse coordination has been assessed and where important methodological gaps remain. A total of 121 evidence sources were included, but only 12 studies employed synchronised measurements of both the rider and the horse based on synchronised time series or shared movement events. Most studies instead examined the rider or horse separately, or focused on individual biomechanical, physiological, or interface measures. Therefore, the primary challenge in this field is not a lack of sensing and measurement technologies, but the absence of a validated and reproducible framework for analysing the rider and the horse together. Future show jumping research should use synchronised measurements, clearly defined jumping events, and standardised coordination measures, while interpreting performance alongside behavioural and clinical information. Such approaches may improve the scientific assessment of performance, safety, and equine welfare.

## 1. Introduction

Equestrian sport combines rider motor skill with the horse’s athletic capacity, making rider–horse coordination a central feature of performance. It can be viewed as a coupled biomechanical interaction [1,2], because performance depends not only on either participant individually but also on the rider–horse dyad [1,2,3]. Studies of rider–horse matching [4] and the concurrent physiological responses in both partners [5] indicate that behavioural, physiological, and welfare-related outcomes are difficult to explain by considering either member of the dyad in isolation [6]. Technologies for the field monitoring of equine welfare and performance have been reviewed separately [7], while evidence on ridden-horse behaviour, welfare, and safety further supports a multidimensional interpretation [8].

From the relational and co-adaptive perspectives, rider–horse coordination is conceptualised here as a continuous reciprocal process in which the rider and the horse adjust to one another, rather than as unidirectional rider control [9,10,11]. The rider posture, weight distribution, centre-of-mass displacement, and movement strategy may influence equine performance and welfare [1]. A review of the rider biomechanics identified the pelvic alignment, dynamic trunk control, and weight distribution as relevant to saddle-pressure patterns and equine gait characteristics [3]. Conversely, the horse’s stride rhythm, force-generation patterns, and responsiveness to aids constrain rider movement. Experimental studies linking the rider and horse motion to the saddle forces [12] and the rein-tension release to the equine response latency [13] illustrate how this interaction may be expressed through movement, interface forces, and timing.

Existing studies have quantified specific components of this interaction, including the rider posture with simplified IMU configurations [14], rider movement during simulated jumping [15], and stirrup forces [16]. These approaches inform the rider movement or interface loading but do not constitute synchronised paired measurements of both the rider and the horse.

Direct dyadic studies using inertial sensing, coordination-dynamics analysis, or synchronisation metrics [17,18,19] demonstrate the feasibility of paired time-series analysis; yet, no universally applicable coordination construct or validated quantitative metric has emerged. Performance models that estimate the rider or rider–horse combination effects [20,21] provide complementary evidence but are conceptually distinct because they are derived from competition records rather than paired movement signals. Much of the literature therefore infers coordination from single-partner, interface, temporal, or aggregate outcomes rather than quantifying it directly. Accordingly, this review distinguishes rider–horse coordination from related constructs such as temporal synchrony, biomechanical coupling, and rider influence.

The three Olympic equestrian disciplines differ in task demands, physical loading, and discipline-specific injury mechanisms [22]. Dressage has the most extensive research base for cyclic rider–horse movement, rider postural control, movement asymmetry, performance assessment, and synchronisation [18,23,24,25,26], together with broader evidence on horse-, rider-, training-, and kinematics-related factors [27]. Eventing contributes pair-level performance evidence, including self- and partner-efficacy [28], although these are not direct biomechanical measures. In show jumping, kinematic and biomechanical variables change rapidly across approach, take-off, flight, and landing [29,30,31], with additional descriptions of back movement and muscle–kinematic relationships [32,33]. The direct synchronised evidence nevertheless remains fragmented; cross-disciplinary findings are therefore used here to identify transferable methodological principles, not to import unvalidated coordination metrics or thresholds into show jumping.

Show jumping is the principal application context because it places brief, phase-dependent demands on the dyad. Performance models can incorporate rider–horse interaction or combination effects [20], and a broader performance analysis links competition outcomes with technical and movement variables [34]. Component studies have described rider effects on equine jumping kinematics [29], back kinematics at take-off [31], and muscle activation during submaximal jumping [32]. Evidence that the head–neck position modifies thoracolumbar and hindlimb kinematics [35] is treated here as a transferable biomechanical principle because it derives largely from cyclic ridden locomotion, not jumping-specific rider–horse coordination.

Biomechanical measurement alone cannot fully characterise the rider–horse interaction; physiological, behavioural, health, and welfare evidence therefore provides important complementary context. Physiological responses vary with exercise, competition context, and experience [5], and equine heart-rate variability depends on the recording and analytical methods [36]. The behavioural indicators of ridden-horse pain should be interpreted with a clinical examination and gait assessment rather than in isolation [37,38]. Studies of rider–horse matching [4] and the concurrent physiological measurement in both partners [5] support including behavioural and physiological information within a broader framework. Biomechanical analysis can inform the interpretation of rider–horse–equipment interactions [2], while evidence from show-jumping horses indicates an association between back pain and reduced competitive performance [39]. These findings do not establish that rider–horse coordination causes health or welfare outcomes.

Recent advances in wearable sensing, sports biomechanics, and artificial intelligence have expanded the scope for objective quantification. IMUs and surface electromyography (sEMG) can provide complementary kinematic and neuromuscular information alongside kinetic, physiological, and behavioural data. Because these systems differ in sampling frequency, placement, measurement purpose, and signal-acquisition principle, paired multimodal measurements require a common time base, cross-device calibration where relevant, consistent event definitions, and appropriate normalisation. A standardised framework for synchronised rider–horse measurement is therefore a methodological prerequisite for testing how coordination-related variables are associated with show-jumping performance, health, and welfare [2,39].

Accordingly, the objectives of this scoping review were as follows:

1. Map the constructs, metrics, and measurement approaches used to assess rider–horse coordination across dressage, show jumping, and eventing, distinguishing direct dyadic measures from rider-, horse-, and interface-specific measures;
2. Compare the three disciplines in terms of measurement domains, data sources, dyadic configurations, and synchronisation methods;
3. Identify transferable methodological principles to guide prospective validation in show jumping, particularly with respect to synchronisation, event definition, and multimodal validation.

## 2. Methods

### 2.1. Study Design and Reporting Framework

A scoping review methodology was selected because rider–horse coordination research is multidimensional, lacks standardised quantitative metrics, and shows substantial heterogeneity in sensor deployment and study protocols. Scoping reviews are designed to map research dimensions and measurement methods, identify knowledge gaps, and delineate future directions without imposing restrictive inclusion criteria [40]. This review was reported in accordance with the Preferred Reporting Items for Systematic Reviews and Meta-Analyses extension for Scoping Reviews (PRISMA-ScR) guidelines [41]. The protocol was not registered in PROSPERO. PROSPERO registration is designed primarily for systematic reviews with a priori defined quantitative synthesis; scoping reviews conducted under PRISMA-ScR guidance are not uniformly required to register, and the present review involved no prospective hypothesis testing or quantitative pooling. Ethics approval was not required because only published sources were used. The evidence map covered the three Olympic equestrian disciplines—dressage, show jumping, and eventing. Show jumping was prespecified as the principal application context rather than the sole inclusion context so that potentially transferable methodological evidence from other disciplines could be considered. Evidence mapping was therefore kept distinct from subsequent show-jumping-specific interpretation.

### 2.2. Search Strategy

Web of Science Core Collection, Scopus, and PubMed were searched systematically; ScienceDirect was used only as an Elsevier platform for full-text retrieval or verification and was not treated as a fourth database or counted independently. Searches were executed on 10 September 2026; the manuscript was subsequently prepared for submission. Given the review’s focus on contemporary quantitative and instrumented methods, publication year was charted as a descriptive evidence-map variable rather than used as an eligibility criterion.

The literature search was structured using the Population–Concept–Context (PCC) framework. The population comprised riders, horses, and rider–horse combinations; the concept included rider–horse coordination, paired measurements or instrument-derived data, performance models, and equine-welfare evidence; and the context covered dressage, show jumping, eventing, and other equestrian settings directly transferable across riding disciplines. Previous work has incorporated rider–horse interaction into show-jumping performance models [20] and explicitly modelled rider effects [21], supporting the inclusion of performance/statistical evidence when synchronised biomechanical time-series data were unavailable. Four complementary search domains were used: (S1) terms explicitly referring to rider–horse coordination, interaction, synchronisation, coupling, synergy, or dyadic relationships; (S2) paired measurements or instrument-derived biomechanical data, including motion capture, inertial measurement units (IMUs), surface electromyography (sEMG), saddle pressure, rein tension, and stirrup force; (S3) athletic performance and statistical or predictive modelling, including rider effects, rider influence, rider–horse combination effects, mixed-effects models, and variance-component approaches; and (S4) evidence on equine welfare. Within S4, welfare- or pain-related factors, mechanical interfaces, behaviour, and physiology were searched separately; stress, rein tension, heart rate, heart-rate variability (HRV), and cortisol were therefore not automatically classified as welfare outcomes.

Search concepts were adapted to the database-specific syntax recorded in File S1. The complete search strings, interface settings, search dates, and the search log are provided in File S1.

### 2.3. Operational Definitions and Inclusion Criteria

In the absence of a universally validated definition or quantitative measure of rider–horse coordination, this review used “evidence of coordination” as the overarching inclusion concept. Not all included studies therefore measured coordination directly. During data charting and evidence synthesis, evidence was classified as (i) direct rider–horse dyadic evidence, (ii) rider-, horse-, or interface-specific evidence relevant to interaction, or (iii) dyad-level performance/statistical evidence. The operational distinctions used in this review are summarised in Table 1.

Eligible publications had to contribute substantively to a conceptual framework, measurement method, or methodological issue relevant to dressage, show jumping, eventing, or a clearly transferable equestrian context. Full-text English publications were eligible if they met at least one of the following criteria: (i) paired measurements of riders and horses; (ii) explicit evidence of rider effects on horse performance or horse effects on rider performance; (iii) saddle, rein, or stirrup interface measurements related to load or signal transfer; (iv) inclusion of variables relating to the rider, horse, or rider–horse combination in a performance model; or (v) evaluation of methods directly applicable to time-synchronised rider–horse analysis. Rider-only or horse-only studies were not classified as dyadic rider–horse coordination studies.

Abstract-only reports, incomplete records, non-English full texts, studies unrelated to Olympic or transferable equestrian contexts, and publications lacking qualifying evidence on interaction, rider effects, interfaces, performance models, or transferable methods were excluded. In addition, 20 publications used for background, methodological support, validation of transferable analytical tools, or interpretive context were designated as contextual/methodological references outside the evidence set and excluded from the evidence denominator, quantitative evidence mapping, figures, and PRISMA inclusion counts. Eligible evidence types included original empirical studies, methodological/technical evaluations, reviews or syntheses, and conceptual/commentary publications that met the operational inclusion criteria. Empirical studies primarily informed observed measures and associations, methodological studies informed feasibility or validity, and reviews or conceptual sources informed established constructs and evidence gaps. Separately, the 20 contextual/methodological references described above were retained only for background or methodological support and were excluded from the 121-source evidence denominator, figures, and PRISMA counts.

### 2.4. Data Charting and Classification

Records were imported into EndNote 2025, and duplicates were removed by comparing DOIs, titles, authors, and publication years, supplemented by manual checks of similar records. Two reviewers independently screened titles and abstracts; disagreements were resolved through discussion, with adjudication by a third reviewer when necessary. Potentially eligible records were retained for detailed eligibility assessment. The reference lists of included studies and relevant background publications were also reviewed to identify additional records. Structured charting forms and tables were used to record key information, including citation details, publication year, evidence type, discipline or context, study objectives and design, reported rider and horse samples, tasks and experimental scenarios, measurement systems, variables, sampling and event definitions, measurement configurations, synchronisation status, outcome types, and main findings.

For quantitative mapping, each empirical or methodological/technical study was assigned to one primary measurement domain: kinematic/biomechanical; kinetic/pressure/interface; neuromuscular/physiological; behavioural/welfare-related; or performance/statistical modelling. Domain assignment reflected the primary outcome rather than every variable reported. Measurement configuration was coded as (i) rider-only, horse-only, or interface-only; (ii) paired rider–horse measurements; or (iii) dyad-level performance/statistical models. A paired study was classified as synchronised only when temporal alignment of rider and horse time series or events was documented through a common time base, trigger, shared event timeline, or equivalent evidence; concurrently acquired data without confirmed temporal alignment were coded separately.

One reviewer led data charting and extracted all predefined fields from every included source into standardised charting forms. A second reviewer verified each entry against the full text and independently reviewed the associated classification decisions. The reviewers’ classifications were then compared, and discrepancies were resolved through discussion; persistent disagreements were adjudicated by a third reviewer.

## 3. Results

**Figure 1:**
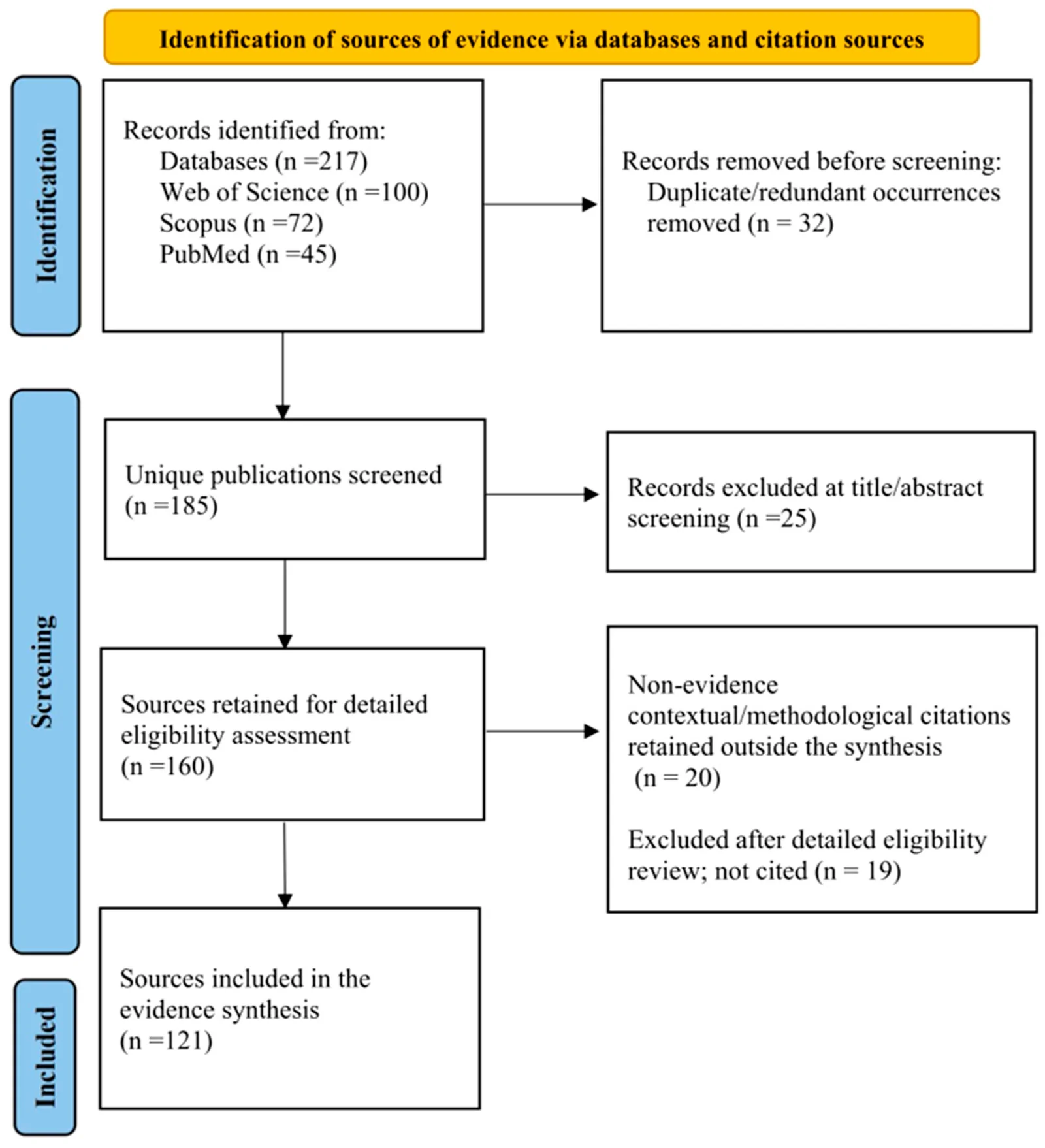
PRISMA-ScR flow diagram of the evidence identification and selection process.

The study selection process is summarised in the PRISMA-ScR flow diagram (Figure 1). Of the 217 records identified, 121 evidence sources were ultimately included in the evidence synthesis.

### 3.1. Evidence Base and Measurement Domains

The included studies primarily addressed dressage or cyclic ridden locomotion (*n* = 42), general or transferable equestrian contexts (*n* = 39), show jumping (*n* = 31), and eventing (*n* = 9). The “general/transferable” category comprised studies whose methods or theoretical frameworks could inform measurement in one or more Olympic equestrian disciplines.

The 121 publications included in the evidence synthesis did not all directly investigate dyadic rider–horse coordination; collectively, they comprised 96 original empirical studies, 12 methodological or technical evaluations, 10 reviews or meta-analyses, and 3 conceptual or commentary publications. The 108 empirical and methodological/technical studies were used primarily to describe measured indicators, associations, and measurement feasibility; the remaining 13 reviews and conceptual/commentary publications supported the theoretical framing and identification of evidence gaps and were not assigned to a primary empirical measurement domain. Across the 108 empirical and methodological/technical studies, five primary measurement domains were identified: kinematic/biomechanical (*n* = 47), performance/statistical modelling (*n* = 23), behavioural/welfare-related (*n* = 15), kinetic/pressure/interface (*n* = 13), and neuromuscular/physiological (*n* = 10).

**Figure 2:**
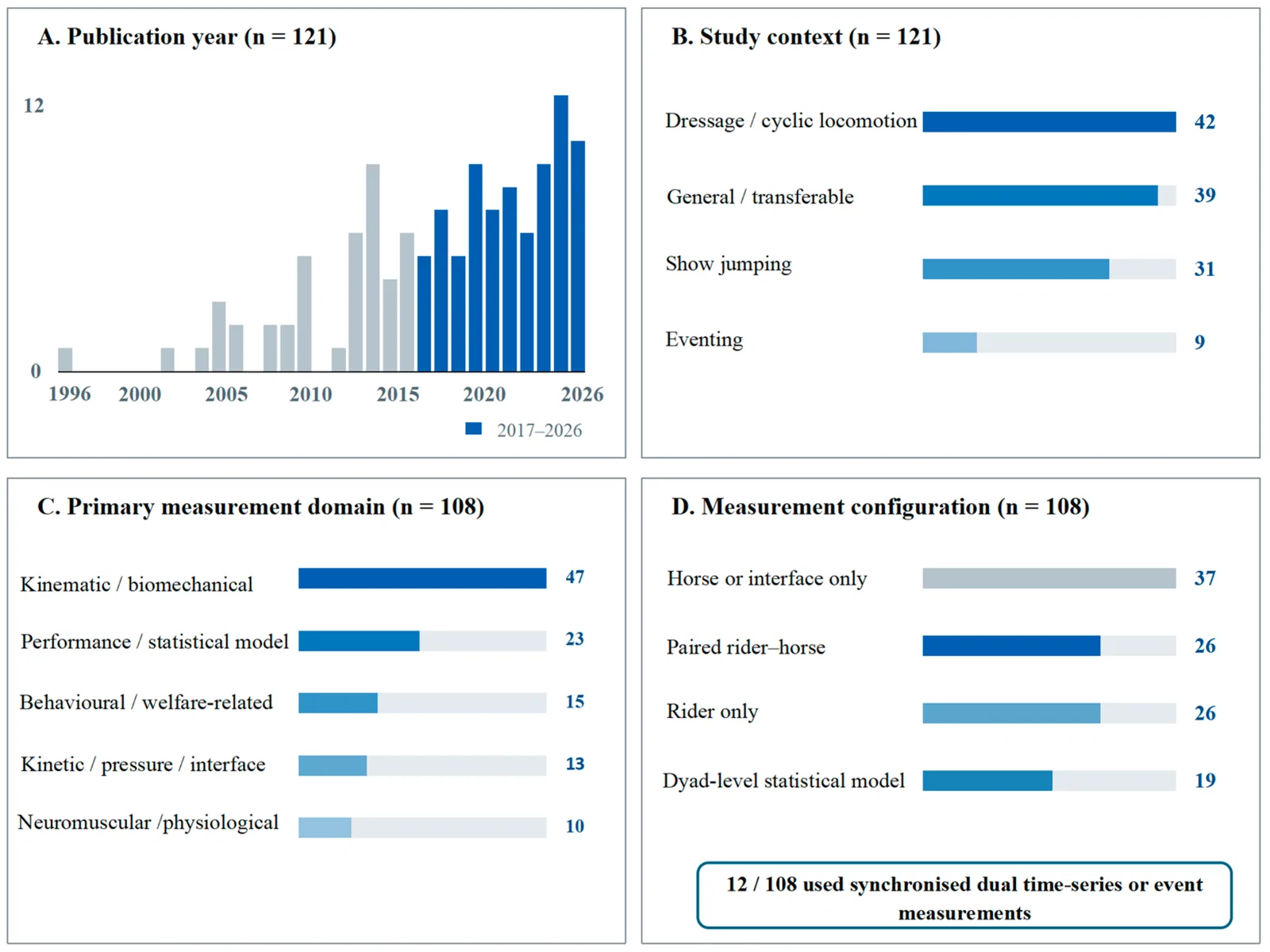
Distribution of the mapped evidence by publication year, study context, primary measurement domain, and measurement configuration: (**A**) publication year and (**B**) study context for all 121 evidence sources; and (**C**) primary measurement domain and (**D**) measurement configuration for the 108 empirical and methodological/technical studies.In panel (**A**), dark blue indicates publications from 2017–2026, whereas light grey indicates earlier publications. In panels (**B**–**D**), different shades are used to distinguish categories visually; the bar lengths and numerical labels indicate the corresponding study counts.

Of these, 37 studies measured horses or rider–horse interfaces without direct paired measurements, 26 focused on riders, 26 included paired measurements of riders and horses, and 19 statistically characterised the dyad in terms of the rider, horse, partnership, or rider–horse interaction effects. Only 12 studies met the strict criterion of synchronised rider–horse time-series or event-based measurements. Paired measurements, dyad-level statistical characterisation, and verified temporal synchronisation were therefore treated as distinct categories. The distribution of the mapped evidence by publication year, study context, primary measurement domain, and measurement configuration is summarised in Figure 2.

#### 3.1.1. Kinematic and Biomechanical Measures

Studies have quantified the equine stride and segment motion, rider pelvic and trunk kinematics, vertical movement symmetry, and sensor-derived performance variables [24,48,49]. These measures characterise the movement timing, posture, trajectory, and symmetry, but variables from one partner should not be interpreted as direct measures of bidirectional rider–horse coordination. Kinematics/biomechanics comprised 47 studies: 17 focused on horses or rider–horse interfaces, 18 on riders, and 12 used paired measurements. Dressage studies examined rider–horse movement relationships under controlled cyclic conditions [26], with direct paired work addressing coordination dynamics, synchronisation, and time-dependent postural regulation [18,19], and inertial-sensor methods developed specifically for rider–horse interaction [17]. Related cyclic work linked equine mouth movements with rider–horse kinematic variables [50], indicating that interaction-related responses may also be expressed through behavioural–mechanical interfaces.

Horse-focused kinematic studies examined limb and back motion, centre-of-mass trajectories, gait symmetry, and jump-related movement characteristics [30,31,51]. Rider-related work reported changes in equine gait parameters associated with rider presence or posture [52], as well as task-dependent rider pelvic and trunk motion and differences in equine movement symmetry or thoracolumbar motion associated with seating style or rider asymmetry [52,53,54]. Studies of cyclic locomotion further examined the circle geometry, gait variability, and auxiliary rein use [55,56,57]. Rider-focused research has also used IMUs to assess the show-jumping technique, rider posture and movement strategies, and performance across skill levels or riding conditions [58,59,60]. Other rider-focused studies have described the postural asymmetry and changes between the active intervention and passive following of the horse’s movement [61,62], the lumbopelvic shock attenuation and coordination associated with the “independent seat” [63,64], and associations of rider physical activity and postural balance with equine welfare indicators [65]. These findings inform rider control and measurement feasibility but remain conceptually distinct from direct paired measurements of rider–horse coordination.

In show jumping, studies have characterised the approach and take-off mechanics, back and limb motion, centre-of-mass trajectories, and muscle–kinematic relationships [29,31,33]. Regnault et al. combined whole-body rider kinematics with bilateral rein-tension and stirrup-force measurements over the final five approach strides [66], while Louis et al. found that a three-IMU rider configuration reproduced key postural measures with a lower setup burden, although centre-of-mass estimates were more variable than with a comprehensive configuration [14].

Wearable IMUs have supported repeated field measurements and gait-event detection [67,68], speed estimation [69], and rider posture monitoring [14], and their broader use in equine gait analysis has been reviewed [70]. Commercial multifunctional inertial sensors have also been used to monitor equine training and movement status [71]. Rider-worn accelerometers have been validated for gait detection, gait and jump classification, and movement-phase segmentation [72,73,74]. The applied work further illustrates the trade-off between measurement completeness and practical deployment. IMUs have been used to quantify saddle-fit-related changes at the rider–horse interface, while open-source wearable platforms have demonstrated the feasibility of real-time riding feedback [75,76]. An automated system for dressage riders has also provided a feasible approach to riding-quality feedback [77].

Kinematic methods remain constrained by task and measurement conditions. High-speed imaging and optical motion capture can provide anatomically interpretable trajectories, but the camera position, marker visibility, and soft-tissue artefacts limit field use. IMU-specific issues, including heading drift, sensor-mounting error, and magnetic interference, may also reduce the comparability across systems [67,69,78]. Movement asymmetry may be present in high-performance horses perceived as non-lame, and rider-perceived laterality may not correspond directly to objective asymmetry [79]. Joint angles, pelvic relative phase, and trunk inclination may therefore serve as task-specific reference variables but should not be treated as universal indicators of rider–horse coordination without validation in an anatomically defined reference frame.

#### 3.1.2. Kinetic, Pressure, and Interface Measures

Kinetic analyses examine the force-related mechanisms underlying kinematic outcomes, focusing on the force transmission and distribution within the rider–horse system. Common measures include ground reaction forces (GRFs), saddle pressure distributions, rein tension, stirrup loading, and other interface forces.

Research on tack has documented changes in facial pressure with noseband tightness and measurable rein, cheekpiece, chain, and intraoral forces in double-rein systems [42,43], suggesting that the equipment configuration may influence the mechanical environment at the rider–horse interface. Studies of back loading have also linked rider and horse movements to loads at the contact interface [80]. Studies of the saddle load distribution have associated rider posture and movement with changes in back loading [81] and quantified differences in saddle and stirrup loads across riding positions [82]; a reduced localised saddle pressure has also been associated with changes in equine gait characteristics [83]. Rider technique, posture, and physical asymmetry may influence back loading and tack load distribution during extended trot [84,85,86]; rider seating position and equipment configuration may affect saddle and stirrup forces and overall rider–horse biomechanics, respectively [82,87]. Of the 13 studies assigned to the kinetics/pressure/interface domain, 8 focused on horse or rider–horse interface measurements, 1 focused on rider measurements, and 4 used paired measurements of riders and horses. This distribution suggests that kinetic data are often used to characterise force transmission within the rider–horse system without directly measuring rider–horse coordination.

Paired measurements of riders and horses, together with tack force measurements, indicate that relationships between movement and load vary with speed [12]. Forces transmitted through specific contact interfaces have also been quantified, including stirrup forces during jumping and rein tension patterns during ridden movement [16,88]. The rein tension and stirrup forces during the approach to an obstacle may vary with rider posture and lead limb position [66]. These measurements are particularly useful for investigating the mechanical load transfer and rider aids, but their interpretation depends on the specific study context.

The rein tension has also been investigated as a candidate mechanical variable for assessing rideability and riding status, suggesting that the same measure may reflect different constructs across experimental contexts [89,90]. Recent studies of tack and nosebands have documented measurable mechanical loading on the face or in the reins, but have not established pressure magnitude alone as an indicator of animal welfare [91]. Accordingly, kinetic and interface measurements can provide mechanistic insight into load transfer, but broader interpretations of animal welfare require the consideration of the biomechanical, behavioural, and clinical context.

#### 3.1.3. Neuromuscular and Physiological Measures

Neuromuscular and physiological measures provide complementary information that cannot be obtained from kinematics alone. Surface electromyography (sEMG), heart rate, heart rate variability (HRV), cortisol, and related measures have been used to characterise muscle recruitment, exercise load, autonomic regulation, and physiological arousal.

Ten studies were included in this domain: one focused on horses or the rider–horse interface, three focused on riders, and six used paired measurements of riders and horses. The concurrent collection of physiological response data does not necessarily imply synchrony between rider and horse movement signals; the relatively high proportion of paired studies should therefore not be interpreted as direct evidence of biomechanical synchrony. Most rider-only sEMG studies have examined trunk muscle activation and measures related to shock attenuation and postural control. Feedback-based interventions have been explored as a means of improving rider trunk control [92], and significant differences in trunk electromyographic activity and shock attenuation have been reported across rider skill levels [93]. Interdisciplinary research has also combined rider kinematics with electromyographic activity under different jumping conditions, illustrating an approach relevant to eventing for the integrated assessment of riders’ lateral biomechanics and neuromuscular function [94].

Studies using paired physiological measurements typically examine horse and rider responses under the same riding or competition conditions. Examples include paired measurements of salivary cortisol in riders and horses during competition [95] and analyses of associations of the horse and rider experience with the equine heart rate, HRV, and cortisol responses during jumping tasks [5]. Similar paired measurements have been used in eventing and show jumping to characterise horse and rider physiological responses under competitive stress [96,97]. Physiological research in show jumping has also examined the associations between riders’ attention and their blood lactate levels [98], as well as paired salivary cortisol measurements in horses and adolescent riders [97]. Physiological responses may also vary with individual rider characteristics, training and competition conditions, and the competition level, emphasising the need for contextual interpretation [99,100,101]. Given the influence of the exercise intensity, sampling timing, weather conditions, analytical methods, and data quality, these variables should therefore be interpreted as context-dependent indicators of the physiological state rather than standalone measures of stress, animal welfare, or rider–horse coordination.

#### 3.1.4. Behavioural and Welfare-Related Measures

Behavioural observations remain important for interpreting the rider–horse interaction and the horses’ responses to ridden work. Research has examined riders’ perceptions of rider–horse harmony, identifying themes that include equine behaviour, rider–horse connection, and rider influence [44,102]. These perceptual constructs, however, are not validated biomechanical measures of rider–horse coordination.

Findings from structured behavioural observations should be interpreted within a broader clinical assessment framework rather than in isolation; research combining ridden exercises with the Ridden Horse Pain Ethogram (RHpE) to inform clinical judgement during pre-purchase examinations of sport horses supports this approach [47]. Of the 15 empirical or methodological studies in the behavioural/welfare-related domain, 9 focused on horses or rider–horse interfaces, 2 focused on riders, and 4 assessed both riders and horses. This domain primarily encompassed aggression, pain and lameness, rider–horse cooperation, temperament, and behavioural responses to riding conditions.

Rider–horse matching has been used to examine the relationships between behavioural and physiological responses [4]; the temperament has also been considered a potential influence on the cooperative behaviour between riders and horses [103]. A recent study quantified conflict behaviour in show jumping [104], and the rider-related variation in equine conflict behaviour, rein tension, physiological measures, and rideability scores has also been quantified [45]. Welfare research in show jumping has examined bit use, while professional assessments of ridden horse behaviour have been compared with physiological measurements [105,106]. These studies provide insight into the mechanisms of behavioural interaction, but do not establish that behavioural consistency or the absence of conflict is equivalent to temporal or biomechanical rider–horse coordination. Research on the relationship between pain and lameness further highlights the need to distinguish empirical welfare evidence from indirect proxy indicators. Studies have compared the behavioural patterns during exercise in horses subjectively assessed as non-lame and those exhibiting lameness [107]. Associations among ridden behaviour, back muscle pain, tack fit, rider posture, and lameness have been reported [108]; evidence from the RHpE and its application to assessing ridden performance in sport horses further supports interpreting these behavioural indicators alongside clinical examinations and the riding context [38,47]. These findings support the clinical interpretation of ridden behaviour but do not, by themselves, establish behavioural manifestations, physiological stress responses, stress levels, or performance errors as valid proxies for animal welfare.

Equine head–neck posture has been associated with conflict behaviour and performance scores [109]. Rider weight, rider–horse saddle fit, and tack configuration are also considered potential modifiers of equine gait and physiological or behavioural responses [110,111,112]. Associations of rider pelvic mobility and balance with equine welfare indicators provide context for considering the rider physical condition when interpreting the rider–horse interaction measures [113]. Integrating validated behavioural observations, clinical examinations, pain or lameness assessments, and the contextual interpretation of physiological data generally provides a more robust basis for welfare assessment than any single proxy measure.

#### 3.1.5. Performance and Statistical Modelling

Because much of the rider–horse interaction literature characterises the dyad through competition outcomes, rider or horse effects, partnership effects, or rider–horse combination effects rather than synchronised biomechanical measurements, performance/statistical modelling was treated as a distinct empirical domain (*n* = 23).

An earlier time–motion analysis and broader performance-analysis research provide baseline approaches for defining the competition structure and candidate outcome variables in quantitative equestrian research [34,114]. In show jumping, the rider–horse interaction has been incorporated into statistical models of sporting performance [20], whereas dressage evaluations have explicitly modelled rider effects in performance data [21]. Repeated competition records and rider–horse effects have likewise been used in quantitative evaluations of sport-horse performance [115,116,117]. In particular, genetic evaluations of show jumping horses have used multilevel competition data, an adjusted fence height, and alternative outcome variables to refine the evaluation of competition performance [118,119,120]. Other performance models have examined relationships involving horse age and the number of riders, as well as competition performance and records of competition experience [121,122,123]. A genetic evaluation has also been applied across show jumping populations, further illustrating the breadth of performance-data-based assessment approaches [124].

At the dyadic level, studies have examined the perceived efficacy within rider–horse pairs [28] and the risk of rider–horse falls during cross-country competition [125]. More recent show-jumping research has applied machine-learning methods to analyse the performance of rider–horse dyads [126]. Rider-centred performance research has also used image analysis as a complementary computational approach to performance assessment [127] and has examined competitive strategies, resilience, and riding experience [128]. In addition, studies have investigated gait-related predictors derived from video analysis, competition data from specific phases of competition, and epidemiological risk factors for falls during cross-country events [129,130,131].

The main contributions, interpretive limitations, and show-jumping relevance of the five measurement domains are summarised in Table 2.

**Figure 3:**
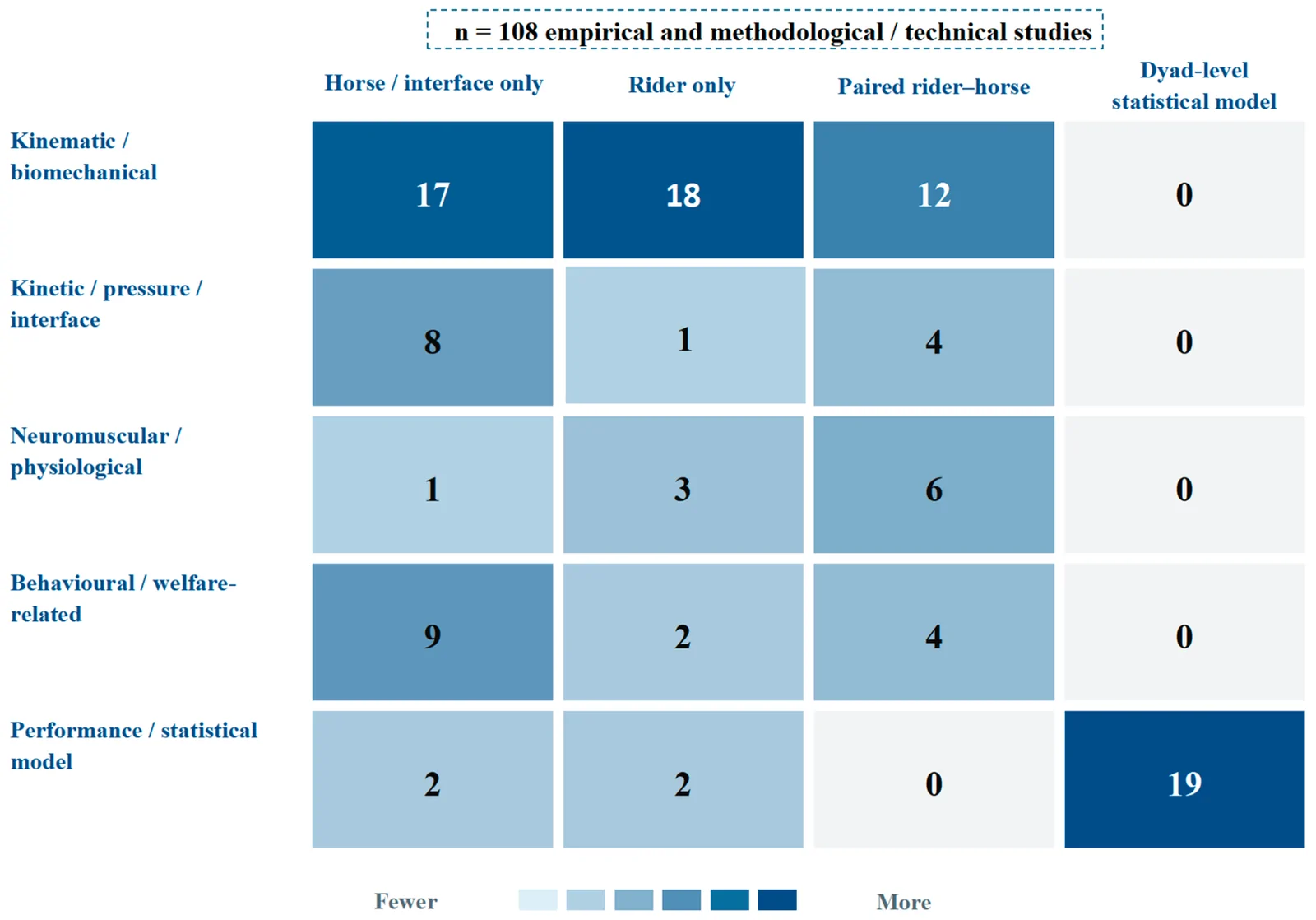
Cross-tabulation of primary measurement domain and measurement configuration among the 108 empirical and methodological/technical studies. Values indicate the number of studies in each category.

Collectively, these studies broaden the evidence base beyond literature explicitly labelled as “coordination” or “synchronisation”, but their interpretation requires a clear conceptual boundary. Rider, horse, combination, and predictive-model effects may identify associations with performance at the rider–horse unit level; yet, they do not by themselves identify the underlying biomechanical mechanisms. Performance/statistical modelling should therefore be regarded as complementary to direct paired measurements of rider–horse coordination rather than as an interchangeable alternative. The distribution of measurement configurations across the five primary measurement domains is shown in Figure 3.

### 3.2. Strengths and Limitations of Current Measurement Approaches

No single measurement approach currently captures all components of rider–horse coordination. Video and optical motion-capture methods provide anatomically interpretable movement data and are useful for identifying and validating biomechanical events; however, their application under competition conditions is constrained by the capture volume, marker visibility, soft-tissue artefacts, and field-deployment requirements. Wearable inertial measurement units (IMUs) partly overcome these spatial constraints and support repeated field measurements, but sensor placement, signal drift, coordinate-system definition, and event-detection algorithms remain important sources of between-study variability. Comparisons between conventional marker-based and markerless approaches further indicate that reducing the measurement burden requires a trade-off between task-specific accuracy and validation requirements; a less instrumented approach should not be assumed to provide an equivalent measurement accuracy across all tasks [133].

Kinetic and interface measurement approaches provide information on mechanical load transfer, but measured forces or pressures should not be interpreted directly as indicators of pain or equine welfare. Neuromuscular and physiological measurements provide complementary information on muscle recruitment, exercise load, and physiological arousal; however, electrode artefacts, data-normalisation procedures, and sampling timing constrain comparisons across studies. Performance/statistical models can quantify the rider, horse, or rider–horse combination effects within competitive performance data [20], but such models do not identify the biomechanical mechanisms underlying those effects. Computational and machine-learning methods should therefore be regarded primarily as analytical tools rather than as independent evidence of a validated rider–horse coordination index. Existing applications demonstrate their feasibility for specific analytical tasks, including a machine-learning analysis of rider–horse dyad performance in show jumping [126]. However, the model performance remains dependent on the dataset, sensor configuration, outcome definition, and validation strategy, limiting the extent to which the findings can be generalised beyond the conditions in which a model was developed and evaluated.

Taken together, the available evidence supports a staged methodological process rather than the adoption of a single universal metric. First, the construct of interest should be defined and individual signals validated against biomechanically meaningful events; validation should report the metric used (e.g., intraclass correlation, root-mean-square error, or detection agreement) and the reference standard against which the agreement was assessed. Second, the rider and horse data should be aligned using a recorded common time base, and the appropriate paired variables should be specified a priori and calculated; reporting should specify the sensor placement, sampling frequency, event definitions, and the temporal-alignment procedure. Finally, these paired variables should be tested to determine whether they provide information beyond that obtained from rider-only, horse-only, or conventional performance measures; incremental value should be evaluated using a predefined criterion, such as a change in the information criterion, cross-validated predictive performance, or an effect size with a stated confidence interval, rather than by statistical significance alone.

### 3.3. Evidence Patterns Across the Three Olympic Equestrian Disciplines

Evidence on the rider–horse coordination across the three Olympic equestrian disciplines is complementary rather than interchangeable. Dressage and other cyclic ridden tasks provide repetitive locomotor conditions that are well-suited to temporal- and phase-based measurements. Show jumping, by contrast, is characterised by non-cyclic and transient demands across the approach, take-off, flight, and landing phases. Eventing adds further complexity through transitions between task contexts, cumulative fatigue, the sequencing of competitive phases, and variation in competition environments. Accordingly, this review adopts a cross-disciplinary comparative approach to identify transferable methodological principles, rather than assuming that variables, thresholds, or coordination patterns derived from one discipline can be directly applied to another.

#### 3.3.1. Dressage and Cyclic Ridden Locomotion

In dressage, riders and horses jointly perform repeated, precisely controlled movements that require a stable rhythm and continuous postural adjustment. Wearable-sensor and biomechanical studies have quantified the rider pelvic motion [48,53], rein tension during transitions and halts [132], relationships between sensor-derived variables and performance assessment, and changes in equine vertical movement under ridden conditions. Visual-search research involving dressage judges further indicates that observational assessment has a task-dependent perceptual structure, reinforcing the distinction between expert judgement and sensor-derived measurement [46].

A key methodological advantage of research in this domain is the repetitive and relatively predictable structure of cyclic locomotion, which facilitates phase-based analysis and repeated observations within the same rider–horse pair. Direct paired studies have characterised coordination dynamics within the rider–horse system [18], examined the rider–horse interaction under ridden conditions [26], and reported the associations between rider position and rider–horse synchrony during a sitting trot [19]. Other dressage-related studies have quantified phase-specific stirrup loading [16], used inertial sensing to assess the rider skill level [58], and demonstrated the feasibility of using field-deployable IMUs to quantify the rider–horse interaction [17].

Accordingly, dressage and other cyclic ridden tasks provide methodological precedents for repeated-event detection, relative-timing assessment, phase-based analysis, and the evaluation of movement repeatability. However, coordination measures or thresholds derived from cyclic locomotion should not be assumed to transfer directly to the non-cyclic, explosive movement sequences characteristic of show jumping.

#### 3.3.2. Show Jumping

Show jumping requires the rider–horse combination to negotiate multiple obstacles while regulating speed, stride organisation, trajectory, and balance while avoiding faults. Unlike cyclic ridden locomotion, the approach, take-off, aerial, and landing phases involve rapid changes in mechanical demands and relatively short time windows for rider–horse adjustment.

Empirical studies have focused primarily on equine kinematics and the effects of the rider presence or riding conditions during obstacle clearance [29,31,51]. Regnault et al. extended the rider-focused evidence by recording the full-body rider kinematics together with bilateral rein tension and stirrup forces during the final approach strides [66]. The rider–horse interaction has also been investigated using simulated jumping tasks [15]. This work provides evidence that multiple component variables can be quantified during simulated jumping; however, a validated integrated metric for assessing synchronised rider–horse coordination throughout the jumping sequence has not yet been established. A further body of evidence in show jumping derives from performance/statistical modelling. The rider–horse interaction or combination effects have been incorporated into quantitative models of show-jumping performance [20], while machine-learning approaches have analysed the rider–horse dyad performance at the outcome level [126]. These approaches broaden the evidence available at the rider–horse combination level, but they should not be interpreted as direct biomechanical measurements of rider–horse coordination.

Accordingly, show jumping is treated as the principal application context in this review because the contrast between increasingly detailed component measurements and the limited integration of direct paired measurements is particularly evident in this discipline, rather than because show jumping already possesses the most established methodology for assessing rider–horse coordination. The methodological transfer to show jumping therefore depends on the integration of clearly defined biomechanical events, synchronised paired measurements, and appropriate outcome validation.

#### 3.3.3. Eventing

Eventing requires the same rider–horse combination to complete the dressage, cross-country, and show-jumping phases. The changing demands across these phases make fatigue, phase sequencing, terrain, speed, and the competition context potentially important modifiers of the rider–horse performance.

High-level competition exercise and fatigue have been associated with changes in stride and jumping characteristics [134], while competition outcomes and safety-related endpoints provide additional information at the level of the rider–horse combination. Eventing safety research has also examined equine fatalities, providing clinically relevant outcome measures; however, these outcomes should be conceptually distinguished from measures used to assess the rider–horse coordination and athletic performance [135]. Eventing research also provides direct examples of treating the rider–horse partnership as the unit of analysis. Self-efficacy and partner efficacy have been examined within eventing rider–horse dyads [28], while epidemiological models have assessed the risk of rider–horse partnerships falling during the cross-country phase [125]. More recent research has further identified the factors associated with the safe completion of cross-country competition [136]. Although these studies have not established a specific biomechanical model of rider–horse coordination for eventing, they highlight two methodological considerations relevant to future show-jumping research: (1) repeated observations should account for the rider–horse pair as the relevant analytical unit; and (2) performance measures may vary with fatigue and with environmental or competition-related conditions. Eventing evidence is therefore particularly useful for identifying ecological moderators and informing longitudinal analyses, but is less likely to provide synchronised coordination metrics that can be transferred directly to show jumping.

The 39 general/transferable sources complement these discipline-specific evidence patterns by providing methodological precedents in sensor validation, rider biomechanics, mechanical interfaces, behavioural analysis, and equine welfare assessment. In this review, their contribution is primarily methodological and conceptual: a technique validated in a general equestrian context is treated as a potential precedent rather than as evidence that the same technique has been formally validated for competitive show jumping. The evidence patterns across the three Olympic disciplines and the general/transferable literature are compared in Table 3.

## 4. Discussion

### 4.1. Implications for Equine Welfare

Rider-related factors may interact with the saddle fit, horse asymmetry, gait characteristics, and pre-existing musculoskeletal conditions [54,108,110], while the training and competition context may modify these relationships [5,134]. Rider balance or asymmetry may affect load distribution, and the head–neck position can modify the caudal back and hindlimb kinematics [35]. These associations provide important context for rider–horse coordination but do not establish a causal link with compromised equine welfare.

Mechanical loading, physiological arousal, behaviour, pain, lameness, and multidimensional welfare are related but non-equivalent levels of inference. The Ridden Horse Pain Ethogram (RHpE) provides a structured framework for recording ridden behaviours associated with musculoskeletal pain [38]. Studies relating ridden behaviour to epaxial pain or hypertonicity, saddle fit, rider position and balance, and lameness [108], together with evidence that the rider skill may influence RHpE-assessed behaviour [137], support interpretation alongside clinical and rider-related context.

Similarly, heart rate, HRV, and cortisol indicate physiological arousal, autonomic regulation, or exercise load rather than welfare directly. Paired rider–horse studies show that experience and competition context can influence these responses [5,95]. The welfare assessment should therefore integrate validated behavioural indicators, clinical examination, pain or lameness assessment, and contextualised physiological data rather than rely on a single surrogate measure.

### 4.2. Translating Cross-Disciplinary Methodological Evidence to Show Jumping

Cross-disciplinary mapping identifies methodological principles that may inform future rider–horse coordination research in show jumping. Dressage and other cyclic ridden tasks provide precedents for repeated-stride measurement, event detection, phase-based analysis, and rider–horse synchronisation [18,19], whereas eventing highlights fatigue, repeated observations, rider–horse pair-level analysis, and ecologically valid competition contexts [28,134]. Because the task structure and movement demands differ substantially among disciplines, variables, analytical frameworks, and decision thresholds should not be transferred to show jumping without discipline-specific validation.

#### 4.2.1. Phase-Specific Coordination Demands in Show Jumping

The jumping effort can be divided biomechanically into the approach, take-off, flight, landing, and recovery phases, although the phase and event definitions vary among studies. During the final approach strides and take-off sequence, the horse regulates the stride organisation, hindlimb propulsion, centre-of-mass trajectory, and trunk–neck configuration, while the rider adjusts the posture, aids, and mechanical interactions through the reins and stirrups.

Recent obstacle-approach research defined the approach as the final five canter strides and identified changes in the rider sagittal-plane kinematics, rein tension, and stirrup loading relative to the seated canter [66]. Other studies quantified stirrup forces during approach, take-off, and landing [16], or examined the rider–horse interaction during simulated jumping [15]. These findings show that the rider-side and interface variables vary across obstacle negotiation but do not establish direct effects on equine biomechanics, performance, or welfare. Terms such as “approach”, “take-off”, and “landing” should therefore be anchored to explicit biomechanical events. For example, studies should distinguish the final approach stride from subsequent hindlimb loading and propulsion, define the final hindlimb ground contact before take-off when that event is analysed, and report the airborne interval and first forelimb ground contact after obstacle clearance where relevant. Explicit definitions would improve the comparability of latency and phase variables.

Event-detection and temporal-alignment approaches from cyclic kinematic research provide useful methodological precedents, but coordination metrics derived from averaged gait cycles should not be assumed to remain valid during the short, non-cyclic transition from approach to take-off. Show-jumping research should therefore prioritise analyses anchored to defined biomechanical events rather than gait-averaged measures.

#### 4.2.2. Implications for Rider and Equine Health

Rider and equine health are important contextual factors when interpreting performance and rider–horse coordination, but the current evidence does not support a simple causal pathway from impaired coordination to injury or pain. Injury-surveillance research confirms clinically relevant risks for riders in competitive equestrian sport [138]. Altered rider trunk control or asymmetry may modify mechanical loading, but such biomechanical changes are not, by themselves, evidence of injury or pain.

Shokry et al. reported poor performance, refusal at jumps, reduced hindlimb impulsion, and back soreness in show-jumping horses with histories of back problems and a reduced competitive ability [39]. This supports an association between equine back problems and reduced jumping performance but does not establish rider loading or rider–horse coordination errors as the cause. Future paired studies should therefore document pre-existing musculoskeletal conditions, tack fit, rider characteristics, and relevant clinical findings to distinguish coordination-related associations from health-, equipment-, or rider-related confounding.

#### 4.2.3. Implications for Jumping Performance

The take-off location and trajectory are important components of obstacle clearance, and Fercher et al. reported take-off distances under defined obstacle and experimental conditions [30]. These values should be treated as condition-specific observations rather than universal optimal thresholds because the obstacle geometry, approach speed, horse characteristics, rider strategy, and other task conditions may influence take-off behaviour.

Powers and Harrison showed that rider condition can alter equine jumping kinematics, including centre-of-mass trajectory, velocity, and spatial positioning [29]. This supports the relevance of rider factors to jumping mechanics but does not establish that a specific rider action causes a particular fault. Performance/statistical studies also associate rider–horse combination effects with competitive outcomes [20] without identifying the underlying biomechanical mechanism. Future work should therefore test whether predefined rider, horse, and paired rider–horse variables are associated with clearly specified outcomes and whether paired measurements add explanatory or predictive information beyond rider-only, horse-only, or conventional performance measures.

### 4.3. Methodological Priorities and Translation to Field Settings

#### 4.3.1. Integration of Wearable and Computational Technologies

Wearable technologies offer practical advantages for collecting repeated measurements outside fixed laboratory capture volumes. IMUs can support field-based measurement of rider posture, equine gait events, and movement timing, while sEMG can provide complementary information on rider or equine muscle recruitment.

The principal methodological requirement is not a greater sensor number or technical complexity, but a clear variable definition, validation for the intended purpose, and reference to a documented common time base. When posture orientation is the primary outcome, a reduced rider-worn IMU configuration may lower the measurement burden [14]. Field-based event-detection studies likewise demonstrate the feasibility of identifying biomechanically relevant events with wearable sensors [67].

Computational methods should be interpreted within the same framework. Explainable artificial intelligence has been applied to equine behavioural classification, while image-analysis and machine-learning approaches have been used for the rider-performance assessment and rider–horse dyad analysis [126,127,139]. These studies demonstrate feasibility for specific analytical tasks but do not validate a general rider–horse coordination score, real-time injury-warning system, or practical refusal-risk threshold.

#### 4.3.2. Multimodal Data Integration and Analysis

A multimodal measurement can capture complementary aspects of the rider–horse interaction: kinematics describe the trajectories and event timing, kinetic/interface measures characterise the load transfer, sEMG reflects the neuromuscular recruitment, and physiological measures provide information on exercise load and arousal. The simultaneous acquisition of these signals, however, does not itself establish a validated rider–horse coordination construct. Each modality should first be validated for its intended outcome, after which the rider and horse data should be aligned to a documented common time base or defined biomechanical event anchors. The reporting should specify the sensor placement, sampling frequency, event definitions, and temporal-alignment procedures. IMU-based event-detection and wearable gait-analysis studies provide methodological precedents for individual components [67,140] but not a validated cross-modal protocol for rider–horse synchronisation.

Once these prerequisites are met, predefined relationships can be examined using statistical methods that account for repeated observations within rider–horse pairs. Correlation and regression analyses, mixed-effects models, and other multilevel models may quantify associations where appropriate, but observational associations should not be interpreted as causal effects. Causal questions require prospective or experimental designs, the explicit consideration of confounding, and methods matched to the study structure and data.

#### 4.3.3. Ecological Validity and Field-Based Validation

Beyond methodological integration, future rider–horse research should extend to authentic training and competition environments rather than relying exclusively on controlled laboratory settings.

Ecological validity is particularly important in show jumping because coordination-related measures may vary with rider experience, horse experience and training level, obstacle type and height, approach speed, repeated efforts, rider–horse familiarity, surface conditions, and competition load. Rider experience has been associated with the differences in equine kinematic and behavioural characteristics [141], while shared rider–horse experience may influence physiological responses during jumping [5]. High-level competition and associated fatigue have also been associated with changes in equine stride and jumping characteristics [134]. Obstacle and task characteristics represent an additional source of variation. Experimental jumping research indicates that biomechanical parameters vary with obstacle configuration [30], suggesting that values obtained during single-obstacle or submaximal protocols should not be assumed to generalise directly to complete competition courses. By contrast, the effects of surface conditions and authentic competition environments on rider–horse coordination remain comparatively underexplored and should therefore be treated as priorities for prospective validation rather than as established modifiers with known effects.

Future studies should consequently evaluate the repeatability and generalisability of coordination-related measures across obstacle types and heights, surfaces, approach speeds, repeated jumping efforts, rider and horse experience levels, rider–horse familiarity, and competition environments. Until such validation has been performed, laboratory-derived values should be regarded as task-specific parameters rather than as universally applicable thresholds or reference values.

#### 4.3.4. From Transferable Methods to Discipline-Specific Validation

Evidence across dressage, show jumping, eventing, and general equestrian contexts remains insufficient for a universally applicable rider–horse coordination framework. Event detection, a common time-base alignment, repeated-measures analysis, and transparent reporting may be transferable, whereas phase relationships, force magnitudes, and performance thresholds require discipline- and task-specific validation. Rider experience, postural control, horse training level, locomotor asymmetry, and rider–horse familiarity should therefore be treated as potential modifiers rather than incorporated into unvalidated composite coordination scores.

The principal evidence gaps and corresponding research priorities for show jumping are summarised in Table 4.

### 4.4. Hypothesis-Generating Roadmap for Rider–Horse Coordination Research in Show Jumping

The current evidence supports the measurement of individual rider–horse system components, but the construct validity, paired-outcome validity, and clinically meaningful welfare or risk thresholds remain unestablished. The pathway below is therefore hypothesis-generating rather than prescriptive: it specifies the order in which validation steps should be undertaken and the reporting elements required at each step, but it does not propose fixed thresholds, universal coordination scores, or causal pathways. Temporal synchrony, biomechanical coupling, rider influence, mechanical load transfer, and behavioural coordination should remain conceptually distinct unless their interrelationships are explicitly tested.

#### 4.4.1. Defining and Validating Paired Quantitative Measures

When the research question concerns direct dyadic coordination, multimodal data should be collected from both the rider and the horse. Obstacle-approach research provides a practical precedent by defining the final approach strides and recording rider kinematics, rein tension, and stirrup forces [66]. A reduced pelvis–sternum–saddle IMU configuration may offer a lower-burden option when the rider posture is the primary outcome [14].

**Figure 4:**
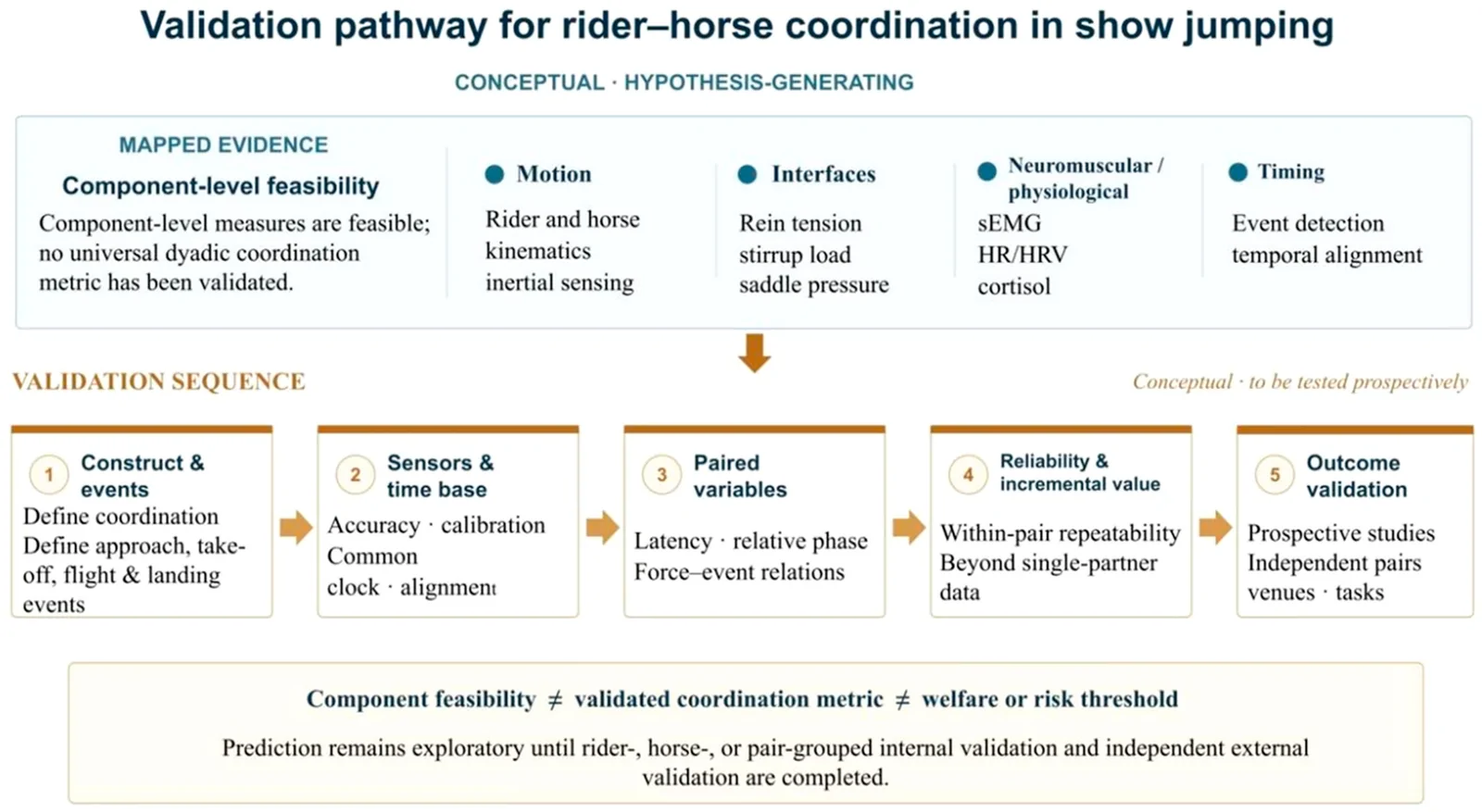
Conceptual and hypothesis-generating validation pathway for rider–horse coordination in show jumping. Component-level feasibility is distinguished from validated coordination metrics and welfare or risk thresholds.

Future paired measurements should align the rider and horse data to a documented common time base and define key biomechanical events before analysis. Candidate variables—including the event-to-event latency, relative phase, movement covariance, rider-aid-to-horse-response latency, and force–event relationships—should be selected according to the coordination construct rather than simply because they are available from sensor outputs.The proposed validation pathway is summarised in Figure 4.

#### 4.4.2. Testing Associations with Performance- and Welfare-Relevant Outcomes

After measurement reliability has been established, predefined rider–horse coordination variables can be examined against clearly defined show-jumping outcomes, including successful jumps, rail contacts or knockdowns, movement trajectories, and take-off- or landing-related variables. Previous studies provide a basis for evaluating the rider, horse, and rider–horse pairing effects [20,21]. Sensor-based research should then determine whether paired measurements provide additional information beyond aggregated or unpaired analyses.

Welfare-related outcomes should be interpreted within a distinct evidential framework. Mechanical load, heart rate/heart-rate variability (HR/HRV), cortisol, and performance errors are not standalone welfare indicators and should be considered alongside biomechanical measures, validated behavioural indicators, and clinical evidence, including a pain or lameness assessment where appropriate [37,38,108]. Correlation and prediction can identify associations but cannot establish that a specific rider–horse coordination pattern causes a particular performance or welfare outcome.

#### 4.4.3. Developing and Validating Predictive Models

Predictive modelling should be considered only after the rider–horse coordination constructs, biomechanical events, measurement reliability, and outcome measures are clearly defined. Machine learning may then evaluate whether predefined multimodal data around key events predict specific performance or welfare-related outcomes. Existing show-jumping work at the rider–horse pair level demonstrates technical feasibility [126] but does not support broadly generalisable real-time prediction. Reliable model development requires a sufficient sample size, separate training, hyperparameter tuning, and independent evaluation data, and partitioning at the horse, rider, or rider–horse pair level to prevent leakage across data sets. External validation should use independent pairs, courses, and task conditions. Validated models could subsequently test whether coordination metrics remain stable or change with repeated jumping, fatigue, course conditions, or competition stage.

## 5. Limitations

This review was restricted to English-language publications, so relevant rider–horse interaction studies in other languages may have been missed. The search was limited to peer-reviewed academic databases and did not systematically retrieve grey literature. Owing to the small number of studies specifically addressing this topic in show jumping, the review remains exploratory in nature, and its conclusions currently lack generalisability; further, more targeted empirical research is needed for validation.

## 6. Conclusions

This scoping review maps the principal constructs, measurement domains, and technologies used in rider–horse coordination research across dressage, show jumping, eventing, and transferable equestrian contexts. The evidence indicates that coordination-related components can be quantified across the kinematic, kinetic/interface, neuromuscular/physiological, behavioural/welfare-related, and performance/statistical domains, but no unified and rigorously validated dyadic framework has yet been established. Two gaps are central for show jumping: direct synchronised paired measurements across the full jumping sequence remain scarce, and standardised procedures for multimodal acquisition, temporal alignment, and integrated analysis are lacking. Transferable methodological principles include explicit event definition, a common time base, repeated-measures analysis, transparent processing, and construct validation, whereas phase relationships, force magnitudes, and performance thresholds require task- and discipline-specific validation.

Future work should therefore prioritise paired rider–horse designs, predefined biomechanical events, and tests of whether paired variables add information beyond rider- or horse-specific measures. Welfare-related outcomes should be interpreted through convergent biomechanical, behavioural, physiological, and clinical evidence rather than any single proxy. Until the construct validity, event definitions, measurement reliability, and outcome validity are established, universal coordination scores, causal welfare claims, and practical risk thresholds are premature. With further validation, the proposed framework may support a more robust investigation of the associations between rider–horse coordination, show-jumping performance, injury risk, and equine welfare.

## Figures and Tables

**Table 1:** Operational definitions used to classify rider–horse coordination-related evidence.

Construct	Operational Meaning in This Review	Typical Measures	Interpretive Boundary
Temporal synchrony	Timing or phase relation between rider and horse signals or predefined events.	Cross-correlation, relative phase, event latency, phase-locking.	Requires a common time base; temporal association alone does not establish directional influence.
Biomechanical coupling	Covariation between movements or forces of the two partners.	Paired kinematics, saddle/rein/stirrup forces, transfer functions.	Coupling can be descriptive; causal interpretation requires an appropriate design.
Rider influence	A change in horse movement, behaviour, physiology, or performance associated with rider identity, posture, aids, or condition.	Rider-condition comparisons, rider fixed/random effects, ridden-versus-unridden contrasts.	Not synonymous with coordination and may be confounded by horse, task, and pairing.
Mechanical load transfer	Force or pressure transmitted through the saddle, reins, stirrups, or ground contact.	Pressure maps and rein tension [42,43]; stirrup force and GRF.	Mechanical exposure is not equivalent to pain, tissue damage, or welfare.
Behavioural harmony	A perceived or behaviourally described quality of the partnership.	Expert ratings, visual attention, conflict behaviour [44,45,46].	Observer-defined harmony is conceptually distinct from objective temporal synchrony.
Welfare evidence	Evidence concerning the animal’s multidimensional physical and mental state.	Validated behaviour, pain/lameness assessment, clinical findings, and contextual physiology [37,38,47].	Heart rate, HRV, cortisol, pressure, or performance errors are not stand-alone welfare outcomes.

Abbreviations: HRV, heart rate variability.

**Table 2:** Measurement domains, contributions, and interpretive limits.

Domain (*n*)	Representative Variables	Contribution	Principal Limitation	Show Jumping Relevance
Kinematic/biomechanical (47)	Stride events, rider pelvis/trunk, back/neck motion, trajectory [19,48,51,66]	Describes movement timing, posture, symmetry, and trajectory.	Many datasets concern one partner; event definitions and coordinate systems vary.	Most developed component domain for approach and take-off.
Performance/statistical (23)	Scores, ranking, rider/horse effects, combination effects, jump outcomes [20,119,128,130]	Quantifies associations and partitions rider, horse, and pairing effects.	Model terms are not direct biomechanical coordination measures; confounding and repeated observations require care.	Useful for outcome definition and hierarchical validation.
Behavioural/welfare-related (15)	Conflict behaviour, harmony, pain/lameness, clinical assessment [4,44,45,111]	Adds horse-centred and clinical context.	Behaviour and arousal are non-specific; welfare requires convergent evidence.	Rarely integrated with jump biomechanics in the same protocol.
Kinetic/pressure/interface (13)	Saddle pressure/force, rein tension, and stirrup load [16,83,88,132]	Characterises mechanical load transfer and aids.	Pressure and force are not equivalent to pain or welfare; field capture can be difficult.	Potential mechanism and validation layer.
Neuromuscular/physiological (10)	sEMG, heart rate, HRV, cortisol [93,94,95,100]	Characterises muscle recruitment, exercise load, and physiological arousal.	Artefact, normalisation, timing, and limited paired evidence constrain comparison.	Potentially useful only when synchronised with movement and interpreted in context.

Abbreviations: sEMG, surface electromyography; HRV, heart rate variability.

**Table 3:** Cross-disciplinary interpretation of the mapped evidence.

Context (*n* = 121)	What the Literature Supports	What Should Not Be Inferred	Transferable Methodological Implication
Dressage/cyclic locomotion (42)	Repeated-stride measurement of pelvis, trunk, symmetry, rein tension, and rider–horse timing is feasible [18,19,48,132].	A universal coordination score or direct transfer of cyclic thresholds to jumping.	Technical precedents for event detection, synchronisation, and repeatability.
Show jumping (31)	Horse jump mechanics, rider effects, and rider-side approach kinematics/interface forces can be quantified [16,30,51,66].	A fixed optimal take-off threshold, validated dyadic index, causal welfare pathway, or current refusal-risk score.	Primary application context and evidence gap.
Eventing (9)	Fatigue, phase-specific performance, partnership variables, and safety outcomes can be studied [125,130,134].	A validated scenario-switching coordination model.	Modifiers and outcome definitions for future longitudinal work.
General/transferable (39)	Sensor validation, rider biomechanics, interface mechanics, behaviour, and welfare constructs can inform protocol design [12,71,83,110].	That a transferable method is already valid for competitive show jumping.	Methodological and conceptual support only.

Evidence counts refer to the mapped evidence base.

**Table 4:** Evidence gaps and proportionate research priorities for show jumping.

Gap	Mapped Pattern	Consequence	Priority
Limited synchronised dyadic measurement	Synchronised precedents are concentrated outside show jumping, including dressage/cyclic studies [18,19] and transferable studies using paired measurements [12,17]; none met the strict definition in the show jumping subset.	Coordination is often inferred from one partner or aggregate outcomes.	Synchronise rider and horse signals and pre-specify paired variables.
Inconsistent events	Approach and limb events vary across protocols [66,67], whereas IMU-derived sensor outputs and computational segmentation are task- and algorithm-specific [68,69,74].	Latency and phase measures are not readily comparable.	Define final approach stride, final hindlimb ground contact before take-off, airborne interval, and first landing contact; report uncertainty.
Weak welfare integration	Physiological and mechanical indicators should not be interpreted as welfare outcomes without behavioural or clinical context [37,38,47], while studies integrating ridden behaviour with clinical, saddle-fit, or rider-related factors illustrate the value of triangulation [47,108].	Risk of equating exposure or arousal with pain or welfare.	Triangulate validated behaviour, pain/lameness, clinical findings, and context.
Limited ecological validity	Controlled or submaximal jumping protocols remain common, while rider experience, rider–horse experience, obstacle/task characteristics, and competition-related fatigue can modify biomechanical or physiological responses [5,30,134,141]. Surface effects and full competition-course conditions remain insufficiently represented in the mapped evidence.	Values or relationships derived under controlled conditions may not generalise across riders, horses, obstacles, surfaces, or competition settings.	Validate proposed coordination measures across obstacle types and heights, surfaces, speeds, repeated efforts, rider and horse experience levels, pair familiarity, and authentic competition environments.
Limited outcome validation	Performance models provide dyad-level or combination-effect precedents [20,21,119]. In the mapped evidence base, explicit paired-versus-single-partner comparisons and external validation were not consistently reported; representative prediction/performance studies include [126,130].	No current basis for practical risk thresholds.	Use horse-, rider-, and pair-grouped validation, independent test sets, repeatability, calibration, and external validation.

Priorities are derived from gaps identified in the mapped evidence.

## Data Availability

No new primary data were generated in this study. All data extracted and analysed during this scoping review are included in the article.

## Supplementary Materials

The following supporting information can be downloaded at: https://www.mdpi.com/article/10.3390/ani16193076/s1, Table S1: Characteristics and classification of sources included in the rider–horse coordination evidence synthesis (*n* = 121); File S1. Database search strategies and search log.
